# 预处理方案含克拉屈滨自体造血干细胞移植巩固治疗急性白血病31例临床观察

**DOI:** 10.3760/cma.j.issn.0253-2727.2021.09.009

**Published:** 2021-09

**Authors:** 圆圆 施, 桂新 张, 祎 何, 明哲 韩, 四洲 冯, 荣莉 张, 尔烈 姜

**Affiliations:** 中国医学科学院北京协和医学院血液病医院（中国医学科学院血液学研究所），实验血液学国家重点实验室，国家血液系统疾病临床医学研究中心，天津 300020 State Key Laboratory of Experimental Hematology, National Clinical Research Center for Blood Diseases, Institute of Hematology & Blood Diseases Hospital, Chinese Academy of Medical Sciences & Peking Union Medical College, Tianjin 300020, China

急性白血病（AL）患者完全缓解（CR）后的巩固治疗方案包括常规化疗、自体造血干细胞移植（auto-HSCT）和异基因造血干细胞移植（allo-HSCT）。高危组成人AL首选allo-HSCT，对于低/中危组AL的巩固治疗方案选择目前尚有争议。auto-HSCT并发症少、移植相关死亡率低、患者移植后生活质量较高，与常规化疗相比，可降低AL患者复发率、改善总生存（OS）率[1]–[3]，故对于缺乏异基因供者的AL患者可选用auto-HSCT进行巩固治疗。与allo-HSCT相比，auto-HSCT存在复发风险高的劣势，降低auto-HSCT后复发率是进一步提高auto-HSCT疗效的关键之一。auto-HSCT后复发的主要原因在于患者体内残存的白血病细胞未被完全清除，因此，优化预处理方案可能是降低auto-HSCT后复发的有效手段之一。克拉屈滨（CLAD）是一种嘌呤核苷类似物，对增殖期和非增殖期的细胞均具有杀伤作用并促进细胞凋亡[4]–[6]，与阿糖胞苷（Ara-C）合用可增强Ara-C的细胞毒性，达到抗肿瘤作用[7]。在复发/难治急性髓系白血病（AML）患者中，CLAD显示了很好的抗白血病作用[8]。我们采用含CLAD预处理方案auto-HSCT用于31例AL患者巩固治疗，取得较好疗效。

## 病例与方法

一、病例

对2016年9月至2020年4月在我院移植中心接受auto-HSCT且符合以下条件的成人AL患者进行回顾性分析：①除外高危组AML；②年龄≥15岁；③移植前疾病状态为CR期；④移植前骨髓及采集物微小残留病（MRD）均阴性；⑤预处理方案中含有CLAD。

移植前CR定义为骨髓白血病细胞<5％、血象基本恢复正常且无髓外白血病[9]。MRD：①采用多色流式细胞术分析（检测敏感度10^−4^），阴性定义为未检测到白血病细胞（<0.01％）；②采用实时荧光定量聚合酶链反应（FQ-PCR）分析PML-RARα、BCR-ABL、AML1-ETO、CBFβ-MYH11、E2A-PBX1、SIL-TAL1等融合基因（检测敏感度10^−5^～10^−6^），阴性定义为FQ-PCR未检测到残留白血病（<0.0032％）。有下列情况之一定义为复发：MRD阳性，形态学复发（骨髓白血病细胞>5％），髓外复发。

二、诊断及危险度分层

所有患者根据细胞形态学、细胞遗传学、分子生物学及免疫表型等检查结果进行诊断，诊断分型标准参照FAB和WHO（2008版）标准[9]–[10]。根据Gökbuget等[11]发表的危险度分组标准对急性淋巴细胞白血病（ALL）进行危险分层；根据2019年美国国家癌症综合网络（NCCN）标准对AML进行危险分层。

三、治疗方案

1. ALL患者的诱导及巩固化疗：所有初诊患者均给予标准VDC（L）P联合方案进行28 d诱导治疗［长春地辛（VDS）4 mg/d第1、8、15、22天；柔红霉素（DNR）45 mg·m^−2^ ·d^−1^第1～3、15～17天；环磷酰胺（Cy）750 mg·m^−2^·d^−1^第1、15天；泼尼松1 mg·kg^−1^·d^−1^第1～14天，0.5 mg·kg^−1^·d^−1^第15～28天；左旋门冬酰胺酶（L-Asp）6000 U·m^−2^·d^−1^第5、8、11、15、18、22天］，Ph染色体阳性（Ph^+^）或BCR-ABL融合基因阳性ALL加用酪氨酸激酶抑制剂（TKI）伊马替尼400～600 mg/d或达沙替尼100 mg/d治疗。获CR后进行巩固化疗，方案包括大剂量甲氨蝶呤（MTX，2 g/m^2^第1天）、VICP方案［VDS 4 mg/d，第1、8天；伊达比星（IDA）10 mg·m^−2^ ·d^−1^，第1～3天；Cy 750 mg/m^2^，第1天；泼尼松1 mg·kg^−1^·d^−1^，第1～14天］、CAM方案［Cy 750 mg·m^−2^·d^−1^，第1、8天；Ara-C 100 mg·m^−2^·d^−1^，第1～3、8～10天；6-巯基嘌呤（6-MP）60 mg·m^−2^ ·d^−1^，第1～7天］等。所有患者诱导缓解后予三联鞘注［MTX 10 mg+Ara-C 50 mg+地塞米松（DEX）10 mg］预防或治疗中枢神经系统白血病，移植前鞘注6（3～7）次。

2. 除急性早幼粒细胞白血病（APL）外AML患者的诱导及巩固化疗：初诊患者采用蒽环类药物（DNR 45 mg·m^−2^·d^−1^第1～3天或IDA 10 mg·m^−2^·d^−1^第1～3天）和Ara-C（100～200 mg·m^−2^·d^−1^第1～7天）方案诱导治疗。获CR后进行巩固化疗，方案包括中、大剂量Ara-C（2～4 g·m^−2^ ·d^−1^第1～3天）±蒽环类药物［DNR 45 mg·m^−2^ ·d^−1^，第1～3天；IDA 10 mg·m^−2^·d^−1^，第1～3天；高三尖杉酯碱（HHT）2.5 mg·m^−2^·d^−1^，第1～7天；米托蒽醌（MTZ）8 mg·m^−2^·d^−1^，第1～3天］、CAG方案［阿克拉霉素7 mg·m^−2^·d^−1^，第1～8天；Ara-C 10 mg·m^−2^·d^−1^，第1～14天；粒细胞集落刺激因子（G-CSF）200 µg·m^−2^ ·d^−1^第1～14天］等。所有患者诱导缓解后予三联鞘注（MTX 10 mg+Ara-C 50 mg+DEX 10 mg）预防或治疗中枢神经系统白血病，移植前鞘注4（1～4）次。

3. APL患者的诱导及巩固化疗：2例初诊APL患者给予全反式维A酸（ATRA）（30 mg·m^−2^·d^−1^）+亚砷酸（ATO 10 mg/d第1～28天）联合Ara-C（100 mg·m^−2^·d^−1^第1～7天）或IDA（8 mg·m^−2^·d^−1^×4 d）诱导治疗，后续以ATO（10 mg/d×21 d或28 d）+ATRA（30 mg·m^−2^·d^−1^）+蒽环类药物（DNR 45 mg·m^−2^·d^−1^，第1～3天；IDA 8 mg·m^−2^·d^−1^，1～4天；MTZ 8 mg·m^−2^·d^−1^，第1～3天）行巩固治疗，并予复方黄黛片、ATRA（30 mg·m^−2^·d^−1^）等维持治疗。复发后予联合化疗，方案包括ATO（10 mg/d×28 d）+IDA（8 mg·m^−2^·d^−1^×4 d）±ATRA（30 mg·m^−2^·d^−1^）及多个疗程包含蒽环类药物（DNR 45 mg·m^−2^ ·d^−1^，第1～3天；IDA 10 mg·m^−2^·d^−1^，第1～3天；HHT 2.5 mg·m^−2^·d^−1^，第1～7天；MTZ 8 mg·m^−2^·d^−1^，第1～3天）联合标准剂量/中剂量Ara-C（100 mg·m^−2^·d^−1^第1～7天，或2 g·m^−2^·d^−1^第1～3天）。

4. 造血干细胞的动员及采集：采用大剂量化疗联合G-CSF动员自体干细胞。ALL患者动员方案：DOAME方案［DEX 10 mg/d第1～5天；VDS 4 mg/d第1天；Ara-C 2 g·m^−2^·d^−1^第1～3天；MTZ 8 mg·m^−2^·d^−1^第2～3天；依托泊苷100 mg/d，第3～5天］，部分患者采用DOAIE动员方案（以IDA10 mg·m^−2^ ·d^−1^第2～3天代替MTZ，余同DOAME方案）。AML患者采用中、大剂量Ara-C（2～4 g·m^−2^·d^−1^第1～3天）联合或不联合蒽环类药物方案动员造血干细胞。患者停化疗后1周开始接受G-CSF 5～10 µg·kg^−1^·d^−1^皮下注射动员造血干细胞，WBC>5×10^9^/L时进行外周血造血干细胞采集。1例患者因外周血造血干细胞数量不足而加行骨髓采集，余30例均采集外周血造血干细胞。所有采集物均进行MRD检测，采集物MRD阴性方可进行auto-HSCT。

5. 预处理方案及回输干细胞数量：所有患者均接受清髓性预处理。12例AML患者的预处理方案：白消安（Bu）3.2 mg·kg^−1^·d^−1^×3 d、CLAD 10 mg/d×3 d、Cy 40 mg·kg^−1^·d^−1^×2 d、Ara-C 2 g·m^−2^·d^−1^×3 d；6例AML患者的预处理未应用Cy，将CLAD和Ara-C疗程加至5 d。13例ALL患者的预处理方案以TBI 3.33 Gy/d×3 d或美法仑50 mg·m^−2^·d^−1^×2 d或依托泊苷20 mg·kg^−1^·d^−1^×2 d代替Bu，其他与AML方案相同。预处理后回输CD34^+^细胞数≥1.5×10^6^/kg。

6. 造血重建及移植后维持化疗：粒系重建定义为中性粒细胞计数≥0.5×10^9^/L连续3 d；血小板重建定义为无血小板输注情况下连续7 d血小板计数≥20×10^9^/L。移植后维持治疗：WBC>3.0×10^9^/L、PLT>50×10^9^/L时，开始1～1.5年的维持化疗，非Ph^+^-ALL患者每2个月1次MM方案（6-MP 60 mg·m^−2^·d^−1^第1～14天；MTX 20 mg·m^−2^·d^−1^第1、8天）与每2个月1次VP/VCP方案（VDS 4 mg/d第1、8天；泼尼松1 mg·kg^−1^·d^−1^第1～14天；Cy 750 mg/m^2^第1天）交替；Ph^+^-ALL患者采用伊马替尼400 mg/d或达沙替尼100 mg/d维持治疗；16例非APL的AML患者中有7例接受维持治疗，包括白介素2（100万U隔日1次）、干扰素（300万U隔日1次）、阿扎胞苷（75 mg·m^−2^·d^−1^×5 d，4周一疗程），1例伴FLT3/ITD突变的AML患者，移植后接受索拉非尼0.4 g/d维持治疗。

四、疗效评估及随访

按Bearman等[12]提出的预处理相关毒性分级标准，在预处理开始至粒系重建进行评分。观察该预处理方案的不良反应，包括非血液学毒性（胃肠道反应、肝肾功能异常、出血性膀胱炎、肝静脉闭塞症、神经系统毒性、感染等）和血液学毒性。

患者于auto-HSCT后定期行骨髓形态、FQ-PCR、多色流式细胞术等评价MRD。所有患者通过门诊、住院病历查阅和电话随访。统计数据包括OS、无白血病生存（LFS）、复发、移植相关死亡、造血重建情况等。OS时间：造血干细胞回输日至死亡或末次随访的时间；LFS时间：造血干细胞回输日至复发、死亡或末次随访的时间；复发时间：造血干细胞回输日至MRD阳性和（或）出现髓外白血病的时间；移植相关死亡：与复发无关的其他死亡。

五、统计学处理

采用SPSS 17.0软件进行统计分析，采用Kaplan-Meier法计算OS率、复发率及LFS率并绘制生存曲线。

## 结果

1. 病例特征：

共有31例AL患者纳入本研究（表1），男21例，女10例，中位年龄33（15～57）岁。起病时中位白细胞计数24.4（1.6～208.0）×10^9^/L。AML患者18例，AML伴重现性细胞学异常5例，分别为AML伴t（15;17）2例、AML伴t（8;21）2例、AML伴inv（16）l例；非特指性AML 13例，其中M_1_ 1例、M_2a_ 5例、M_4_ 1例、M_5_ 6例。14例检出FLT3-ITD、C-kit、NPM1、CEBPA、IDH2、TET-2、NRAS、NOTCH2等突变。非APL的AML按2019年NCCN指南细胞遗传学分组，预后中等组7例，预后良好组9例。ALL患者13例，其中Ph^+^ B-ALL5例，T-ALL 1例，B-ALL 7例；染色体及分子生物学检测结果显示，2例染色体核型正常，5例t（9;22）或BCR-ABL阳性，1例t（1;19）/E2A-PBX1阳性，1例SIL-TAL1阳性，4例不详。2例检出TET2、SETD2、CREBBP、KMT2D等突变。ALL预后分组：高危组10例，标危组3例。除2例APL为CR_2_，其余29患者均为CR_1_，所有患者移植前MRD检测均阴性，诊断至移植的中位时间为8（5～12）个月，移植前化疗中位疗程5（3～9）个。

**表1:** 31例接受含克拉屈滨预处理自体造血干细胞移植巩

项目	结果
中位年龄［岁，*M*（范围）］	33（15～57）
性别［例（％）］	
男	21（67.7）
女	10（32.3）
疾病诊断［例（％）］	
ALL	13（41.9）
高危组	10（32.3）
标危组	3（9.7）
APL	2（6.5）
AML（排除APL）^a^	16（51.6）
良好组	9（29.0）
中等组	7（22.6）
初诊染色体核型［例（％）］	
正常	13（41.9）
异常	9（29.0）
未见核分裂相	2（6.5）
不详	7（22.6）
移植前病程^b^［月，*M*（范围）］	8（5～12）
单个核细胞回输量［×10^8^/kg，*M*（范围）］	9.01（2.06～20.97）
CD34^+^细胞回输量［×10^6^/kg，*M*（范围）］	2.10（1.59～10.44）
粒细胞植入时间［d，*M*（范围）］	12（9～34）
血小板植入时间［d，*M*（范围）］	19（10～113）

注：ALL：急性淋巴细胞白血病；APL：急性早幼粒细胞白血病；AML：急性髓系白血病。^a^按NCCN遗传、分子生物学预后分组；^b^除外2例APL患者（27、47个月）

2. 预处理相关毒性及移植相关并发症：

预处理期间，发生轻度胃肠道反应26例、中度胃肠道反应1例及轻度肝损害9例，予保肝、护胃等治疗后好转。粒系重建前发生血流感染5例、肺感染2例、肠道感染5例、肛周感染8例、口腔感染7例、甲沟炎2例，予积极抗感染治疗后好转。未发生出血性膀胱炎、肝静脉闭塞症及神经系统毒性；无肾功能异常病例及移植相关死亡病例。采用含CLAD的预处理方案均达到清髓效果。

3. 回输自体干细胞的数量及造血重建：

回输单个核细胞的中位数为9.01（2.06～20.97）×10^8^/kg，CD34^+^细胞的中位数为2.10（1.59～10.44）×10^6^/kg。所有患者均获得造血重建，中性粒细胞重建的中位时间为12（9～34）d，血小板重建的中位时间为19（10～113）d。

4. 生存分析：

随访至2020年11月，中位随访时间为18.5（7.5～50.0）个月，31例患者中30例存活，1例因复发死亡。预期2年OS率为（94.7±5.1）％，LFS率为（80.1±7.3）％，累积复发率为（19.9±7.3）％。

共有6例（19.4％）患者移植后复发，复发中位时间为4.5（0.5～10）个月；6例复发患者中，ALL、AML各3例；3例为血液学复发，其余3例为MRD阳性。1例血液学复发的AML患者予FLAG方案［氟达拉滨（Flu）30 mg·m^−2^·d^−1^，第1～5天；Ara-C 1 g·m^−2^·d^−1^，第1～5天；G-CSF 200 µg·m^−2^·d^−1^，第0～5天］联合化疗未缓解，后予输注血制品、抗感染等支持治疗，移植后18个月死亡；2例血液学复发（ALL、AML各1例）患者接受allo-HSCT治疗，获得完全血液学缓解及完全供者嵌合状态并获得长生存；3例（ALL 2例，AML 1例）移植后MRD阳性患者予维持化疗后MRD转阴，具体治疗：1例AML患者采用白介素2、干扰素交替维持治疗，1例Ph^+^-ALL患者采用达沙替尼100 mg/d维持治疗，1例非Ph^+^-ALL患者采用VP（VDS +泼尼松）与MM（6-MP+MTX）方案交替维持治疗。

31例AL患者中，≤2个疗程联合化疗后MRD转阴患者（27例）的预期2年LFS率优于>2个以上疗程MRD转阴性患者（4例）（84.7％对50.0％，*P*＝0.057）；在非APL的16例AML患者中，≤2个疗程联合化疗后白血病MRD转阴患者的LFS率显著优于>2个以上疗程后MRD转阴患者（3例）（92.3％对33.3％，*P*＝0.005）。

## 讨论

虽然auto-HSCT后复发率高，但移植相关死亡率低、感染并发症低、移植后患者生活质量较高及不受供者来源限制，故对于缺乏HLA相合供者的MRD阴性的AL患者来说，auto-HSCT仍是巩固治疗选择之一。近年的文献报道显示CR1期的AML、尤其是非高危组CR1期的AML及标危组ALL患者、获得首次完全分子学缓解的Ph^+^-ALL患者行auto-HSCT与allo-HSCT的疗效相当[13]–[19]。Mizutani等[14]回顾性研究1276例成人CR_1_期AML患者，其中375例行auto-HSCT、521例行异基因骨髓移植（allo-BMT）、380例行异基因外周血干细胞移植（allo-PBSCT）治疗，5年OS率分别为65％、62％、55％，5年LFS率分别为60％、59％、51％。auto-HSCT组5年OS率优于allo-PBSCT组（*P*＝0.004），与allo-BMT组差异无统计学意义（*P*＝0.422）；auto-HSCT组患者的5年LFS率与allo-BMT组、allo-PBSCT组差异无统计学意义（*P*值分别为0.759、0.145）。Giebel等[16]回顾性分析569例获得首次完全分子学缓解的成人Ph^+^-ALL患者，67例行auto-HSCT、255例行同胞供者allo-HSCT、247例行无关供者造血干细胞移植（URD-HSCT）治疗，auto-HSCT组2年OS率、LFS率与allo-HSCT组、URD-HSCT组差异均无统计学意义（*P*值分别为0.58、0.69）。另外Chakrabarty等[20]多中心回顾性研究294例CR2期行造血干细胞移植APL患者，发现auto-HSCT组疗效优于allo-HSCT组。而对于ALL患者而言，通过加强auto-HSCT后维持化疗可进一步降低复发率、改善预后[21]–[22]。Powles等[21]通过前瞻性研究发现，auto-HSCT后行维持化疗可提高疗效，10年OS和无病生存（DFS）率分别达53％和50％，10年复发率为42％；同时研究发现，auto-HSCT后接受2或3种药物维持治疗的患者与未接受维持治疗或接受1种药物维持治疗的患者比较，复发率显著降低（*P*＝0.004），DFS率（*P*＝0.0005）和OS率（*P*＝0.0008）显著提高。

需要注意的是，auto-HSCT因缺乏移植物抗白血病效应，移植后复发的风险增高。如何降低移植后复发，是改善auto-HSCT疗效的关键。研究显示，AL患者auto-HSCT前MRD是影响移植后OS、LFS的独立预后因素，auto-HSCT前MRD阴性患者的OS、LFS明显优于MRD阳性患者[23]–[27]。另外，我们的研究也发现，31例AL患者中，2个疗程以内白血病MRD转阴性患者的LFS优于3个或3个以上疗程白血病MRD转阴的患者（*P*＝0.057）；在非APL的16例AML患者中，2个疗程以内白血病MRD转阴性患者的LFS显著优于3个或3个以上疗程白血病MRD转阴性的患者（*P*＝0.005）。因此，这些研究显示，化疗后MRD越快越早转阴性的患者，自体移植疗效越好。

改良预处理方案、加强预处理的抗肿瘤作用，进一步清除患者体内残存的白血病细胞，也是降低移植后复发的有效方法。CLAD是一种嘌呤核苷类似物，对增殖期和非增殖期的细胞均具有杀伤作用；可通过间接抑制DNA甲基化转移酶，消耗甲基供体而发挥去甲基化作用，进而促进细胞凋亡[4]–[6]。另外CLAD与Ara-C合用时可增加细胞对Ara-C摄取，使细胞内Ara-C含量增加，增强Ara-C的细胞毒性、提高抗肿瘤作用[7]。CLAD常见的不良反应主要是骨髓抑制、免疫抑制及感染，治疗后注意加强口腔、肛周护理以降低感染风险。Holowiecki等[8]前瞻性随机对照研究652例成人初治AML患者，患者中位年龄47（17～60）岁，将患者随机分成三组，分别采用标准DA方案、标准DA方案+CLAD、标准DA方案+Flu方案进行诱导治疗，三组患者的CR率分别为51％、62％、55％；三组患者缓解后的治疗方案相同，中位随访2.8年，3年OS率分别为33％、45％、35％；此研究证实，CLAD组患者的CR率和OS率得到显著改善。Bao等[28]研究发现，在103例难治复发AML患者中，分别选用FLAG（Flu+Ara-C+G-CSF）或CLAG（CLAD+Ara-C+G-CSF）方案化疗，CLAG组患者的CR率（61.7％对48.7％）与3年OS率（35.3％对26.8％）优于FLAG组患者。

基于以上研究，对于无HLA相合供者的31例MRD阴性的AL患者，我们采用含CLAD、Ara-C的改良预处理方案行auto-HSCT，作为患者获得CR后的巩固治疗方案。预处理期间非血液学毒性主要为轻中度胃肠道反应、肝功能损伤及感染，均在3级以下，予积极治疗后均好转，无移植相关死亡病例；血液学毒性方面，预处理后所有患者均为Ⅳ级骨髓抑制，达到清髓的目的，移植后所有患者均获得造血重建，中性粒细胞、血小板重建的中位时间分别为12 d、19 d，与文献[27],[29]–[30]报道一致。随访至2020年11月，1例AML自体移植后复发患者接受联合化疗后未缓解，于移植后18月死亡，其余30例患者均存活。存活患者中，有25例患者移植后MRD持续阴性，5例移植后复发患者予维持化疗或allo-HSCT治疗后，MRD转阴性；预期2年OS率为（94.7±5.1）％、LFS率为（80.1±7.3）％、复发率为（19.9±7.3）％，总体疗效优于文献[27],[29]–[31]报道。本组患者疗效较好可能与以下因素相关：①研究患者中，除2例APL患者为CR2，其余患者均为CR1，且所有患者移植前MRD均阴性；②我们在预处理方案中加入CLAD，可进一步清除患者体内残存的白血病细胞，从而降低移植后复发的风险；③对于ALL患者，我们在移植后加用TKI类药物或VP+MM方案等维持治疗，降低了移植后复发率，从而提升了总体疗效。当然，本研究亦存在一些不足之处，如病例数较少、非随机对照研究、随访时间短等。另外，9例预后良好组AML患者中，2例患者移植后接受维持治疗，3例患者移植后复发；而7例预后中等组AML患者中，5例患者移植后接受维持治疗，2例患者因血象恢复缓慢尚未接受维持治疗，所有患者无白血病复发。我们分析，AML患者auto-HSCT后的维持治疗可能降低移植后复发风险。

综上，对MRD阴性、无HLA相合供者的AL患者，尤其是2个疗程以内白血病MRD转阴性的患者，使用含CLAD预处理方案，增强了预处理的抗白血病作用，同时未增加预处理相关毒性，移植后OS率、LFS率较高，可作为AL患者auto-HSCT的有效预处理方案。
